# Arbuscular Mycorrhizal Fungi and Nutrition Determine the Outcome of Competition Between *Lolium multiflorum* and *Trifolium subterraneum*

**DOI:** 10.3389/fpls.2021.778861

**Published:** 2021-12-23

**Authors:** Stephan Unger, Franziska M. Habermann, Katarina Schenke, Marjan Jongen

**Affiliations:** ^1^Department of Experimental and Systems Ecology, University of Bielefeld, Bielefeld, Germany; ^2^MARETEC—Marine, Environment and Technology Centre, LARSyS, Instituto Superior Técnico, Universidade de Lisboa, Lisbon, Portugal

**Keywords:** arbuscules, grass, interactions, legume, mycorrhiza, soil nutrients, symbiosis, vesicles

## Abstract

Arbuscular mycorrhizal fungi (AMF) may affect competitive plant interactions, which are considered a prevalent force in shaping plant communities. Aiming at understanding the role of AMF in the competition between two pasture species and its dependence on soil nutritional status, a pot experiment with mycorrhizal and non-mycorrhizal *Lolium multiflorum* and *Trifolium subterraneum* was conducted, with manipulation of species composition (five levels), and nitrogen (N)- and phosphorus (P)- fertilization (three levels). In the non-mycorrhizal state, interspecific competition did not play a major role. However, in the presence of AMF, *Lolium* was the strongest competitor, with this species being facilitated by *Trifolium*. While N-fertilization did not change the competitive balance, P-fertilization gave *Lolium*, a competitive advantage over *Trifolium*. The effect of AMF on the competitive outcome may be driven by differential C-P trade benefits, with *Lolium* modulating carbon investment in the mycorrhizal network and the arbuscule/vesicle ratio at the cost of *Trifolium*.

## Introduction

Grasslands are important ecosystems, as they provide a major food source, while preserving plant and animal diversity and offering additional ecosystem services, such as resource storage, soil protection, and recreational opportunities (White et al., 2000; Unger and Jongen, 2015). Population growth together with increasing environmental change impacts on grassland performance (Unger and Jongen, 2015; Knapp et al., 2017; van Oijen et al., 2018) has accentuated the need for a sustainable solution to improve grassland productivity, which has led to changes in traditional grassland management in many countries. In the Iberian Peninsula, sowing legume-rich seed mixtures for the establishment of permanent pastures (the so-called SBP, Sown Biodiverse Pastures) is one example (Teixeira et al., 2015). Seed mixtures of highly productive legumes and grasses are introduced in semi-natural pastures in order to increase soil nitrogen availability and improve productivity (Teixeira et al., 2015). This practice has been successful, with studies showing enhanced productivity in SBP, as compared to semi-natural pastures (Carneiro et al., 2008; Hernández-Esteban et al., 2019), thereby significantly increasing the sustainable stocking rates and providing animals with forage of higher nutritional value and plant digestibility. However, sustainability of SBP may be threatened by various factors, such as drought (Jongen et al., 2013, 2019), overgrazing (Sales-Baptista et al., 2016), or phosphorus limitation. Legumes and associated *Rhizobium* fix atmospheric nitrogen, making the system self-sufficient in nitrogen; yet an external input of phosphorus for optimal legume growth is necessary to sustain the positive effects of SBP (Teixeira et al., 2015). In addition, the efficacy of SBP may be affected by competition between grasses and legumes, which in turn can depend on the availability of macronutrients, mainly nitrogen and phosphorus. A high nitrogen supply increases the competitive strength of grasses, as rapid growth increments strengthen their ability to compete with other species for water and light (Silvertown et al., 2006; Song et al., 2011; Hacker et al., 2015). In turn, nitrogen addition has been shown to inhibit legume biomass and abundance, with the competitive strength of legumes generally being promoted by phosphorus rather than nitrogen addition (Ren et al., 2017) because of rhizobial symbiosis allowing for autonomous nitrogen acquisition (Beringer et al., 1979). Thus, phosphorus addition may be one of the key factors enhancing the abundance of legumes and their ability to promote grass growth by nitrogen input into the system, with the soil nitrogen and phosphorus stoichiometry being determinant for positive long-term effects and sustainability of the SBP practice.

One important mediator of soil nutrient acquisition by plants is the arbuscular mycorrhizal fungal (AMF) symbiosis. Although some studies showed AMF to be significant in enhancing plant nitrogen supply (Hodge and Fitter, 2010; Ashgari and Cavagnaro, 2012; Fellbaum et al., 2012; Whiteside et al., 2012), AMF are mainly known to increase phosphorus uptake (Koide et al., 2000; Smith and Smith, 2011), with many plant species (including the legumes in the SBP) being obligately dependent on phosphorus supply through mycorrhizal symbiosis (Janos, 2007). As is the case for plant performance in general, the nutritional benefits of mycorrhiza to a plant may depend strongly on soil N:P stoichiometry, with the possibility of lower benefits or even detrimental effects of mycorrhiza at low soil N:P ratios (Johnson, 2010). Although plants are able to control mycorrhizal colonization to a certain degree (Friede et al., 2016, 2017), carbon allocation toward mycorrhiza under low mycorrhizal phosphorus benefits may strongly decrease the productivity in mycorrhizal plants as compared to non-mycorrhizal plants (Smith and Smith, 2013). In addition, at low soil nitrogen, the mycorrhizal fungi can become a competitor for nitrogen to the plant (Unger et al., 2016) and it may also affect the competitive interactions between different plant species through the development of common mycelial networks (CMNs) interlinking plants with different carbon investments or nutrient demand from the AMF (Merrild et al., 2013; Fellbaum et al., 2014; Höpfner et al., 2015; Montesinos-Navarro et al., 2019). Finally, both N and P transfer to the plant may be a direct consequence of the fungal demand for nutrients (Hodge et al., 2010).

Insights into mycorrhizal functioning and plant-fungal processes are important for understanding the competitive interactions between the grasses and legumes in SBP, and their potential role in the sustainability of these pastures. The predominant species used in the SBP mixture are the legume, *Trifolium subterraneum* and the grass, *Lolium multiflorum*. Both functional groups not only have different nutrient demands (Hill et al., 2005; McCaskill et al., 2019), but also have different nutrient acquisition strategies, with grasses commonly being facultatively mycotrophic (Reinhart et al., 2012) and relying on their large and highly branched fine root system to acquire nutrients, while many legumes show a strong nutritional dependency on mycorrhiza (Hempel et al., 2013). As AMF can deliver phosphorus more efficiently than the root uptake pathway (e.g., Smith et al., 2011), the highly mycorrhizal legume, *T. subterraneum* is therefore expected to gain a competitive advantage over *L. multiflorum*, particularly under low soil P conditions. A previous study comparing grassland species belonging to the functional groups of forbs, grasses, and legumes showed that phosphorus benefits increase with larger investments in mycorrhiza, while nitrogen benefits increase with a larger investment in roots, leading to a trade-off in carbon allocation between mycorrhizal and root structures (Unger et al., 2016), which may be crucial for nitrogen and phosphorus competition between legumes and grasses under diverse soil N:P stoichiometry. Increasing the competitive ability, and hence the relative abundance, of legumes in sown pastures can be pivotal to achieving sustainability and continued nitrogen fixation with long-term productivity increases in SBP systems. This study contributes to this aim by investigating mycorrhizal effects on the competitive ability of the prominent grass and legume in SBP with different N- and P- fertilization. A pot experiment with mycorrhizal and non-mycorrhizal *L. multiflorum* L. and *T. subterraneum* L. was conducted, with the manipulation of species composition (five levels) and N- and P-fertilization (three levels). We hypothesized that (i) AMF would promote the competitive ability of the legume (i.e., *T. subterraneum*) over the grass (i.e., *L. multiflorum*), and that (ii) the influence of AMF on competitive outcome would be markedly modulated by N- and P-fertilization.

## Materials and Methods

### Experimental Design

A controlled pot experiment was conducted in a greenhouse at the University of Bielefeld, Germany. The experiment followed a randomized complete block design with three factors: with and without mycorrhizal inoculum (AM/NM), five competition treatments (competition 1–5), and three fertilizer regimes (C, +N, +P), with five replicates for each treatment, giving a total of 150 pots. In the competition treatments, we assessed the individual responses of two species typically dominant in the SBP seed mixtures: the legume, *T. subterraneum* L. and the grass, *L. multiflorum* L. The species composition in the five competition treatments is depicted in Supplementary Figure 1. Competition 1 is comprised of four individuals of *Trifolium*, competition 2 contains three *Trifolium* individuals and one *Lolium* individual, competition 3 contains two individuals of *Trifolium* and two individuals of *Lolium*, competition 4 contains one *Trifolium* individual and three *Lolium* individuals, and competition 5 is comprised of four individuals of *Lolium*. All competition treatments were subjected to three different fertilizer regimes with either standard nutrient solution (C), increased nitrogen (+N), or increased phosphorous (+P).

### Plant Material and Cultivation

Seeds of *T. subterraneum* L. and *L. multiflorum* L. (Genyen, Grow and Protect Lda., Lisbon, Portugal) were surface-sterilized (70% ethanol for 1 min, rinsed with the mixture of 2% NaOCl and 1 drop of 32% HCl for 10 min) and sown in boxes with sterilized (120°C for 1.5 h) sand. Three weeks after germination, the seedlings were transplanted to small pots (0.5 l) filled with sterilized sand. During transplanting, roots of AM treatment seedlings were inoculated using 20 g of an inoculum-sand-mixture containing dried *Plantago lanceolata* root fragments with at least 200,000 infective units of *Rhizophagus irregularis* per liter inoculum (INOQ GmbH, Schnega, Germany), while the NM treatment received 20 g of sterilized sand and 5 ml of a microbial wash, which was extracted from the inoculum by sieving the supernatant of a water-inoculum-mixture through a 20 μm sieve (Koide and Li, 1989). Pre-inoculation was necessary to ensure that all plants were mycorrhizal and viable during the experiment. After 4 weeks, the plants were transplanted again, this time into 2 l-containers using a template depicted in Supplementary Figure 1 to ensure equal distances between the four plants in a pot. Each pot received a nutrient package with 3 g of a solid long-term fertilizer containing 20% N, 15% K, 10% P, 6% Mg, 2% S, 0.112% Mn, 0.09% Cu, 0.057% B, 0.05% Fe, 0.025% Zn, and 0.008% Mo (PlantoSan, Hauert MANNA Düngerwerke GmbH, Nürnberg Germany). These packages were made of a 40 μm mesh, so that nutrients were only accessible for mycorrhiza but not for plant roots. However, results showed that nutrients from the packages were only provided through diffusion, with equal distribution among all treatments, regardless of mycorrhization. The purpose of extra nutrients from the package to be only available for mycorrhizal plants was thus not fulfilled and will not be explored further.

Plants were cultivated for another 7 weeks in a greenhouse at a photosynthetic photon flux density of ∼400 μmol m^–2^ s^–1^, a temperature of ∼19°C, and a relative humidity of ∼60%. Pots were watered daily with deionized water and twice a week, all pots received 30 ml of a modified Hoagland nutrient solution (Hoagland and Arnon, 1950) depending on the respective fertilizer treatment, i.e., control, +N or +P (Supplementary Table 1). Pots were randomized weekly to ensure equal growth conditions. At an age of 14 weeks, all plants were harvested and separated into above- and belowground materials. After cleaning the roots from the substrate, all plant materials were oven-dried at 60°C for 72 h and weighed. Rhizobial nodulation of *Trifolium* roots was observed across all treatments but was not quantified. Soil samples of ∼70 g were taken and dried at 40°C for further analysis of soil P. In addition, soil samples of ∼15 g were frozen at −20°C for the analysis of soil N.

### Quantification of Mycorrhizal Root Colonization

Representative subsamples of the extracted roots of both AM and NM plants were analyzed for the quantification of mycorrhizal colonization. The roots were bleached in 10% KOH at 90°C for 10 min, rinsed with deionized water, and stained with an ink-acetic-acid solution (1:1:8 = ink: 10% acetic-acid: H_2_O) at 90°C for 15 min (Vierheilig et al., 1998). The root fragments were then transferred to microscope slides and the percentage of root length colonized by AMF was estimated at 250 times magnification using a modified intersect method (McGonigle et al., 1990), scoring a minimum of 100 intersections per sample for the presence of hyphae, vesicles, and arbuscules. The A:V ratio, indicative of the C–P trade between the symbionts [i.e., organic carbon (C) used by the fungi and phosphorus (P) delivery] was calculated as the ratio of the fraction of root length containing arbuscules to the fraction containing vesicles.

### Quantification of Tissue Nitrogen and Phosphorus

Foliage of dried aboveground plant material was ground in a ball-mill (Retsch MM 301, Retsch, Haan, Germany) prior to further analysis. Subsequently, 2–4 mg of ground plant material was transferred to an elemental analyzer (EuroVector, HEKAtech, Wegberg, Germany) and analyzed for total elemental C and N. Tissue P was measured using high-temperature oxidation and colorimetrical quantification according to Watanabe and Olsen (1965). Dried aboveground plant material was ashed at 500°C for 4 h in a muffle furnace and, after cooling, 2–4 mg of ash was digested in 10% nitric acid. The extracts were diluted with bidistilled water and analyzed for orthophosphate concentration using flow injection analysis at 880 nm (FIA-Lab II, MLE GmbH, Dresden, Germany).

### Quantification of Soil Nitrogen and Phosphorus

Soil NO_3_^–^ and NH_4_^+^ were extracted by shaking 15 g of soil with 30 ml of 0.1 M CaCl_2_ for 1 h.

Soil PO_4_^3–^ was extracted using a modified calcium acetate–lactate (CAL) extraction method according to Schüller (1969). A suspension of 4 g of soil and 30 ml of CAL solution (77 g calcium lactate, 39.5 g calcium acetate, and 89.5 ml 100% acetic acid l^–1^) was shaken for 90 min. Extracts of both mineral nitrogen and phosphorus were centrifuged at 3,000 rpm for 3 min and the supernatant was passed through a glass fiber filter (1 μm pore size) using a Luer syringe. Concentration of N and P in the extracts was determined by colorimetric analysis at 546 and 880 nm, respectively, using flow injection analysis (FIA-Lab II, MLE GmbH, Dresden, Germany).

### Data Analyses

For each species, at a given treatment, mycorrhizal growth dependency was calculated according to Eq. 1 (Smith et al., 2003):


(1) $$\text{MGD}\left(\%\right)=100\times \left(\text{AM}-\bar{\text{NM}}\right)/\text{AM}$$

with AM corresponding to the total biomass of an individual mycorrhizal plant and corresponding to the mean total biomass of the equivalent non-mycorrhizal plants.

For each species at a given treatment, mycorrhizal P-dependency was calculated according to Eq. 2 (Smith et al., 2003):


(2) $$\text{MPD}\left(\%\right)=100\times \left({\text{AM}}_{\text{P}}-\bar{{\text{NM}}_{\text{P}}}\right)/{\text{AM}}_{\text{P}}$$

with AM_*P*_ corresponding to the tissue P (%) of an individual mycorrhizal plant and corresponding to the mean tissue P (%) of the equivalent non-mycorrhizal plants.

Total aboveground biomass was used to calculate the relative neighbor effect (RNE) according to Eq. 3 (Markham and Chanway, 1996; Callaway et al., 2002):


(3) RNE=(Bcontrol-Bmix)/x

with x being B *control* if B *control* > B *mix* and x being B *mix* if B *control* < B *mix*, where B *mix* is the aboveground biomass of a plant in interspecific competition (competition 2, 3, and 4) and B *control* is the aboveground biomass of an intraspecific control (competition 1 for *Trifolium* and competition 5 for *Lolium*).

Tissue P (%) of plant material was used to calculate the relative neighbor effect for P-competition (RNE-P) according to Eq. 4 (Markham and Chanway, 1996; Callaway et al., 2002):


(4) RNE-P=(Pcontrol-Pmix)/x

with x being P *control* if P *control* > P *mix* and P *mix* if P *control* < P *mix*, where P *mix* is the tissue P (%) of a plant in interspecific competition (competition 2, 3, and 4) and P *control* is the tissue P (%) of an intraspecific control (competition 1 for *Trifolium* and competition 5 for *Lolium*).

The relative performance ratio (PR) of the two species in the mixture at a given treatment is calculated according to Eq. 5:


(5) $$\text{PR}={\text{B}}_{Lolium}mix/{\text{B}}_{Trifolium}mix$$

with B *mix* corresponding to the aboveground biomass per plant of the two species in interspecific competition (competition 2, 3, and 4).

Statistical analyses were performed using Statistica 6.0 (StatSoft Inc., Tulsa, OK, United States). Data were tested for normal distribution (Kolmogorow-Smirnow test) and homogeneity of variances (Brown-Forsythe test). Data that did not satisfy the assumptions of normal distribution was log10 or arcsine square root transformed prior to analysis. Two-way ANOVA (factors: competition, fertilization) was performed on the data of colonization level, MGD, MPD, and A:V ratio within each species. Three-way ANOVA (factors: competition, mycorrhization, and fertilization) was performed on above- and belowground biomass, RNE, RNE-P, tissue N and P concentration of each species, soil N and P, and on PR. When appropriate, Tukey’s *post hoc* pairwise comparison was applied to determine the individual differences between means.

## Results

### Mycorrhization

**FIGURE 1:**
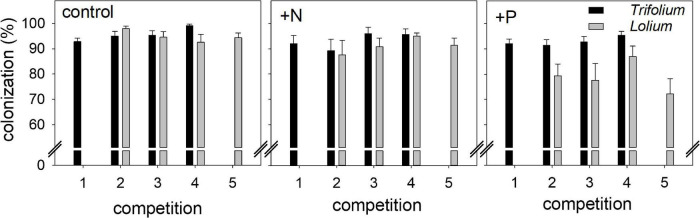
Mycorrhizal root colonization (%) in *Trifolium subterraneum* (■) and *Lolium multiflorum* (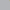), subjected to standard fertilization (control), enhanced N-fertilizer (+N) or enhanced P-fertilizer (+P), in the five competition treatments. Data represent mean ± standard error, *n* = 5.

Mycorrhizal inoculation led to a high AMF root colonization in both species (Figure 1). In *Trifolium*, root colonization ranged from 89.2% (+N, competition 2) to 99.2% (control, competition 4), with a significant effect of competition on *Trifolium* root colonization (Table 1). In *Lolium*, fertilization significantly affected root colonization, with significantly lower colonization levels in the +P treatment (average value of 79.1%) as compared to the control and +N treatment (average value of 93.1%).

**TABLE 1:** Results of two-way ANOVA for factor competition (1–5) and fertilization (control, +N, +P) on mycorrhizal colonization, A:V ratio and MPD of *Trifolium subterraneum* and *Lolium multiflorum*.

	Mycorrhizal colonization		A:V ratio		MGD		MPD	
	*Trifolium*	*Lolium*	*Trifolium*	*Lolium*	*Trifolium*	*Lolium*	*Trifolium*	*Lolium*
Competition (C) (1, 2, 3, 4, 5)	**0.048** b, b, ab, a, –	0.40	0.66	**0.026** –, a, ab, ab, b	0.15	**0.012** –, b, a, ab, a	**0.001** a, ab, b, b, –	**0.014** –, a, ab, ab, b
Fertilization (F) (control, +N, +P)	0.21	**<0.001** a, a, b	0.10	**0.040** a, b, a	0.57	**0.022** a, ab, b	0.54	**<0.001** a, b, b
C × F control (1, 2, 3, 4, 5) +N (1, 2, 3, 4, 5) +P (1, 2, 3, 4, 5)	0.84	0.37	0.99	0.59	0.66	**<0.001** –, ab, abc, a, a –, c, a, a, a –, ac, ac, bc, ab	0.10	0.88

*Data stated are P-values, with significant effects (P < 0.05) given in bold. Different letters for factor competition, fertilization, or the interaction of competition × fertilization indicate significantly different means (Tukey’s HSD post hoc, P < 0.05).*

**FIGURE 2:**
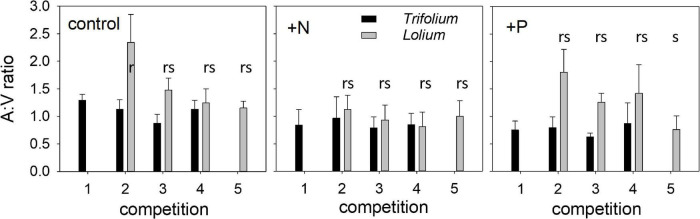
Ratio of the percentage root length containing arbuscules to vesicles (A:V ratio) in *T. subterraneum* (■) and *L. multiflorum* (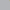), subjected to standard fertilization (control), enhanced N-fertilizer (+N) or enhanced P-fertilizer (+P), in the five competition treatments. Data represent mean ± standard error, *n* = 5. Different letters (r–s) indicate significantly different means (Tukey’s HSD *post hoc*, *P* < 0.05) for factor competition in *L. multiflorum*.

The ratio of arbuscules to vesicles (A:V ratio) was higher in *Lolium* than in *Trifolium* (Figure 2). In *Lolium*, both competition and fertilization significantly affected the A:V ratio (Table 1). Values in the control and the +P treatment were significantly higher as compared to the + N treatment. In addition, in the control and the +P treatment, the highest A:V ratio was found in those pots having only one *Lolium* individual.

### Biomass

**FIGURE 3:**
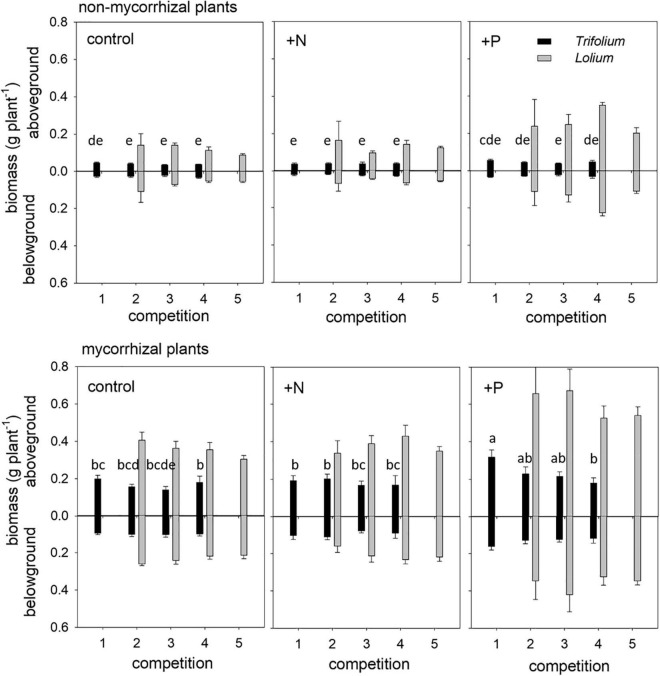
Above- and belowground biomass of non-mycorrhizal (NM) and mycorrhizal (AM) *T. subterraneum* (■) and *L. multiflorum* (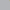), subjected to standard fertilization (control), enhanced N-fertilizer (+N) or enhanced P-fertilizer (+P), in the five competition treatments. Data represent mean ± standard error, *n* = 5. Different letters (a–e) indicate significantly different means (Tukey’s HSD *post hoc*, *P* < 0.05) for factor competition for aboveground *T. subterraneum*.

The overall competition significantly affected the aboveground biomass of *Trifolium* (Table 2), with a decreasing trend of *Trifolium* biomass per plant with increasing presence of *Lolium* (Figure 3). This trend was significant for mycorrhizal individuals in the +P treatment (Figure 3). There were neither significant competition effects on the aboveground biomass of *Lolium*, nor on belowground biomass of either species (Table 2 and Figure 3), although there was a trend of increasing *Lolium* biomass with increasing presence of *Trifolium* in both the control and the +P treatments.

**TABLE 2:** Results of three-way ANOVA for factor competition (1–5), mycorrhization (NM, AM), and fertilization (control, +N, +P) on above- and belowground biomass of *T. subterraneum* and *L. multiflorum*, and the performance ratio (PR).

	Aboveground biomass		Belowground biomass		PR ratio
	*Trifolium*	*Lolium*	*Trifolium*	*Lolium*	
Competition (C) (1, 2, 3, 4, 5)	**0.024** a, ab, b, ab,-	0.34	0.56	0.21	0.65
Mycorrhization (M)	**<0.001**	**<0.001**	**<0.001**	**<0.001**	**0.019**
Fertilization (F) (control, +N, +P)	**0.002** b, b, a	**<0.001** b, b, a	**0.003** ab, b, a	**<0.001** b, b, a	**0.042** b, b, a
C × M	0.10	0.76	0.49	0.58	0.97
C × F	0.33	0.98	0.77	0.20	0.84
M × F	**0.017**	0.19	**0.018**	0.49	0.39
NM (control, +N, +P)	c, c, c		c, c, c		
AM (control, +N, +P)	b, b, a		b, b, a		
C × M × F	0.46	0.46	0.79	0.21	0.90

*Data stated are P-values, with significant effects (P < 0.05) given in bold. Different letters for factor competition, fertilization, or the interaction of mycorrhization × fertilization indicate significantly different means (Tukey’s HSD post hoc, P < 0.05).*

Mycorrhizal inoculation positively affected above- and belowground biomass in both species (Table 2 and Figure 3). The strongest positive growth effects of AMF were found in *Trifolium*, with 4.7- and 4.1-fold higher above- and belowground biomass in mycorrhizal individuals, as compared to non-mycorrhizal individuals, respectively. In *Lolium*, growth stimulation due to the inoculation was 2.8- and 3.2-fold for above- and belowground biomass, respectively. The differential growth responses of the two species to mycorrhization is reflected in the PR ratio (Tables 2, 3), with significantly higher values in the non-mycorrhizal pots (average of 5.3 ± 0.84) as compared to the mycorrhizal pots (average value of 3.1 ± 0.32).

**TABLE 3:** The performance ratio (PR) of the two-species mixture subjected to standard fertilization (control), enhanced N-fertilizer (+N) or enhanced P-fertilizer (+P), in the competition treatments 2, 3, and 4.

Fertilization (F)	Competition (C)	NM	AM
Control	2	3.78 ± 1.70	2.76 ± 0.49
	3	4.27 ± 0.56	2.80 ± 0.47
	4	3.13 ± 0.61	2.23 ± 0.41
+N	2	5.71 ± 3.88	1.93 ± 0.54
	3	3.03 ± 0.77	2.57 ± 0.46
	4	4.00 ± 0.94	4.16 ± 1.65
+P	2	7.83 ± 5.89	3.78 ± 1.54
	3	7.14 ± 2.11	3.43 ± 0.85
	4	8.32 ± 1.45	3.77 ± 1.45

*Data represent mean ± standard error, n = 5.*

Fertilizers significantly affected the above- and belowground biomass in both species (Table 2 and Figure 3). Biomass was not affected by nitrogen addition. However, phosphorus addition significantly enhanced the above- and belowground biomass in both species, as compared to the control and the +N treatment (Table 2), with the exception of belowground biomass for *Trifolium*, where significant differences were only observed between the +N and +P treatment. The growth stimulation due to phosphorus addition was higher in *Lolium* than in *Trifolium*, with significantly higher PR values in the +P addition (Tables 2, 3), both for non-mycorrhizal and mycorrhizal pots (average of 5.7 ± 1.10), as compared to the control and the +N treatment (average of 3.2 ± 0.34 and 3.6 ± 0.71, respectively).

### Tissue Nutrients

**FIGURE 4:**
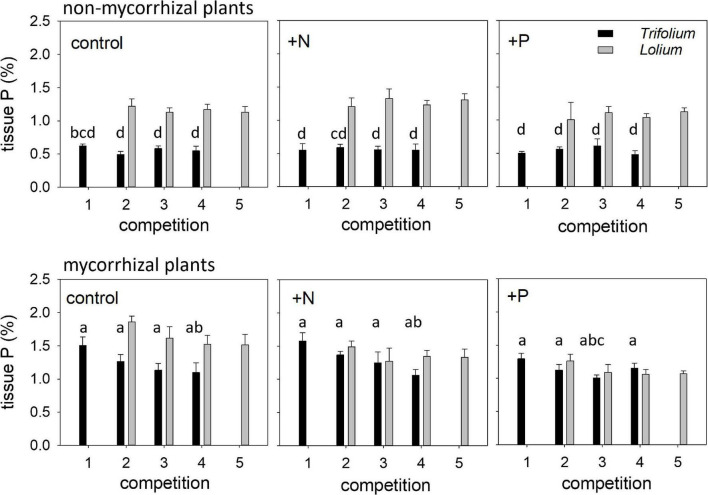
Tissue phosphorus (%) of NM and AM *T. subterraneum* (■) and *L. multiflorum* (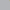), subjected to standard fertilization (control), enhanced N-fertilizer (+N) or enhanced P-fertilizer (+P), in the five competition treatments. Data represent mean ± standard error, *n* = 5. Different letters (a–d) indicate significantly different means (Tukey’s HSD *post hoc*, *P* < 0.05) for factor competition in *T. subterraneum*.

Tissue P concentration differed between the two species (Figure 4). In the non-mycorrhizal plants, tissue P concentration in *Lolium* was more than double than in *Trifolium*. In the mycorrhizal plants, higher tissue P concentration in *Lolium*, as compared to *Trifolium*, was only found in the control. Competition significantly affected tissue P concentration in *Trifolium* (Table 4 and Figure 4). In the mycorrhizal plants, highest tissue P concentration was found in competition 1, i.e., in those pots with four *Trifolium* individuals, while the opposite trend was observed in *Lolium*, i.e., lower tissue P concentration with increasing number of *Lolium* individuals.

**TABLE 4:** Results of three-way ANOVA for factor competition (1–5), mycorrhization (NM, AM), and fertilization (control, +N, +P) on tissue phosphorus, tissue nitrogen, RNE and RNE-P of *T. subterraneum* and *L. multiflorum*.

	Tissue P (%)		Tissue N (%)		RNE		RNE-P	
	*Trifolium*	*Lolium*	*Trifolium*	*Lolium*	*Trifolium*	*Lolium*	*Trifolium*	*Lolium*
Competition (C) (1, 2, 3, 4, 5)	**0.028** a, ab, ab, b, –	0.36	0.83	0.73	0.35	0.21	0.30	0.47
Mycorrhization (M)	**<0.001**	**<0.001**	**<0.001**	0.48	0.10	0.87	**<0.001**	0.09
Fertilization (F) (control, +N, +P)	0.25	**<0.001** ab, a, b	0.62	0.13	**0.003** ab, b, a	0.11	**0.033** a, ab, b	0.36
C × M	0.07	0.19	0.86	0.82	0.74	0.21	0.48	0.14
C × F	0.79	0.88	0.42	0.41	0.93	0.53	0.86	0.95
M × F NM (control, +N, +P) AM (control, +N, +P)	0.72	**0.001** bc, bc, c a, b, bc	**<0.001** bc, ab, a ab, c, c	**0.01** ab, ab, ab a, ab, b	0.78	0.64	0.32	0.78
C × M × F	0.58	0.99	**0.033**	0.36	0.74	0.15	0.45	0.94

*Data stated are P-values, with significant effects (P < 0.05) given in bold. Different letters for factor fertilization, or the interaction of mycorrhization × fertilization indicate significantly different means (Tukey’s HSD post hoc, P < 0.05).*

Mycorrhization significantly affected tissue P concentration in both species, this effect being more prominent in *Trifolium*, with average values for tissue P concentration of 0.56 and 1.23% in the non-mycorrhizal and mycorrhizal individuals, respectively. Fertilization significantly affected tissue P concentration in *Lolium*, with the lowest tissue P in the +P treatment.

**FIGURE 5:**
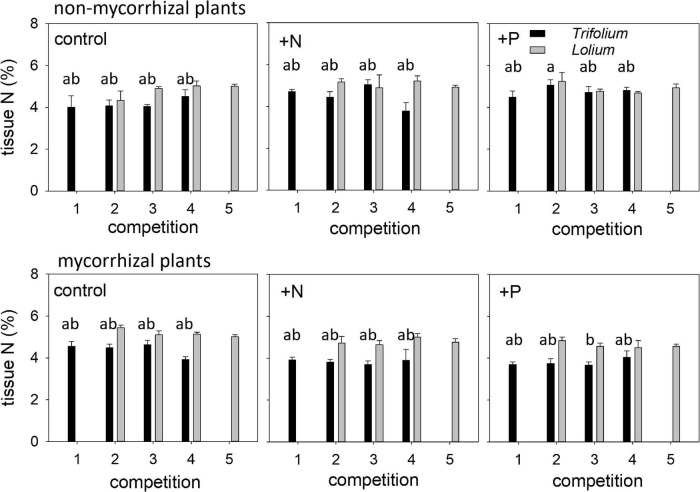
Tissue nitrogen (%) of NM and AM *T. subterraneum* (■) and *L. multiflorum* (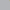), subjected to standard fertilization (control), enhanced N-fertilizer (+N) or enhanced P-fertilizer (+P), in the five competition treatments. Data represent mean ± standard error, *n* = 5. Different letters (a, b) indicate significantly different means (Tukey’s HSD *post hoc*, *P* < 0.05) for factor competition in *T. subterraneum*.

Tissue N concentration in both species was neither affected by competition nor fertilization (Table 4 and Figure 5). Mycorrhization only affected tissue N concentration in *Trifolium*, with significantly lower tissue N in the mycorrhizal plants for the +N and +P treatment, as compared to the non-mycorrhizal plants.

### Mycorrhizal Dependency and Neighbor Effects

**FIGURE 6:**
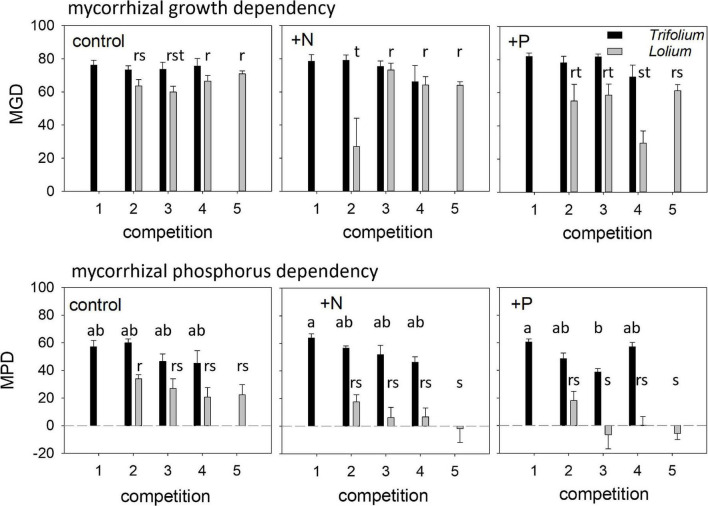
Mycorrhizal growth dependency (MGD) and mycorrhizal phosphorus dependency (MPD) of *T. subterraneum* (■) and *L. multiflorum* (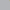), subjected to standard fertilization (control), enhanced N-fertilizer (+N) or enhanced P-fertilizer (+P), in the five competition treatments. Data represent mean ± standard error, *n* = 5. Different letters (a–b for *T. subterraneum* and r–s–t for *L. multiflorum*) indicate significantly different means (Tukey’s HSD *post hoc*, *P* < 0.05) for factor competition.

In *Trifolium*, mycorrhizal growth dependency (MGD) was not affected by competition or fertilization, while both factors did affect MGD in *Lolium* (Table 1), although a clear trend in the latter species was absent (Figure 6). Mycorrhizal P-dependency (MPD) was significantly affected by competition in both species (Table 1), with a decreasing MPD with increasing presence of *Lolium* (Figure 6). In addition, in *Lolium*, fertilization led to a significantly higher MPD in the control as compared to the +N and the +P treatment (Table 1 and Figure 6). Marked differences in MPD between the two species can be observed, with a higher P-dependency in *Trifolium* (average of 52.7 ± 2.20) as compared to *Lolium* (average of 11.5 ± 3.9).

**FIGURE 7:**
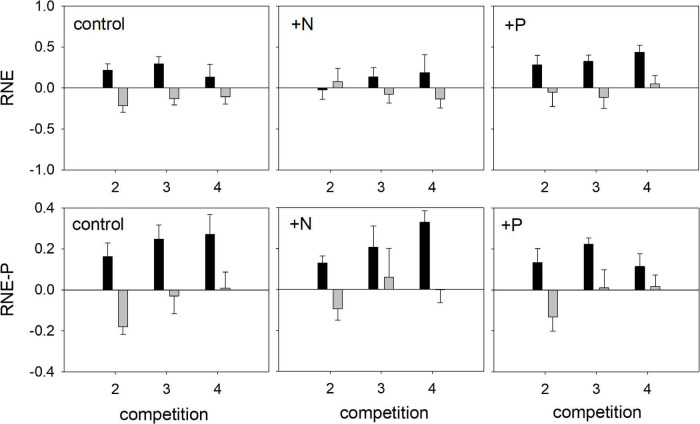
Relative neighbor effect (RNE) and relative neighbor effect for P-competition (RNE-P) of mycorrhizal (AM) *T. subterraneum* (■) and *L. multiflorum* (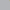), subjected to standard fertilization (control), enhanced N-fertilizer (+N) or enhanced P-fertilizer (+P), in the competition treatments 2, 3, and 4. Data represent mean ± standard error, *n* = 5.

Regarding biomass accumulation, the relative neighbor effect (RNE) for both species was not affected by competition or mycorrhization (Table 4). Fertilization did affect RNE for *Trifolium*, with significantly higher values in the +P treatment, as compared to the +N treatment (AM plants, Figure 7 and NM plants, Supplementary Figure 2). The relative neighbor effect for P- acquisition (RNE-P) of *Lolium* on *Trifolium* was significantly affected by mycorrhization and fertilization (Table 4). However, the presence of *Trifolium* did not result in significant RNE-P factor effects on *Lolium*. Regarding RNE-P, clear trends are obvious in the mycorrhizal plants (Figure 7), with *Trifolium* being subjected to competition, while *Lolium* was facilitated. This contrast intensified with the increasing presence of the other species.

### Soil Nutrients

Competition did not affect soil inorganic P, although mycorrhization and fertilization did result in significant differences in soil P (Supplementary Table 2). In the non-mycorrhizal pots, soil P was significantly higher as compared to the mycorrhizal pots (Supplementary Table 3). In addition, not surprisingly, soil P was significantly higher in the +P treatment, as compared to the control and the +N treatment.

Total soil inorganic N was significantly affected by competition (Supplementary Table 2), with soil N decreasing from competition treatments 1–5, i.e., with increasing presence of *Lolium* (Supplementary Table 4).

## Discussion

### Effects of Arbuscular Mycorrhizal Fungi on Competitive Balance Between *Trifolium* and *Lolium*

The majority of available literature supports the relevance of a high degree of mycotrophy for enhanced competitive strength (Hetrick et al., 1994; Crush, 1995; Smith et al., 1999; Scheublin et al., 2007), but there are also contrasting studies (Hodge, 2003; Daisog et al., 2012; Höpfner et al., 2015). We hypothesized that AMF would promote the competitive ability of the legume (i.e., *T. subterraneum*) over the grass (i.e., *L. multiflorum*), as phosphorus uptake *via* the AMF pathway should be more efficient than *via* the direct root pathway (Smith et al., 2011) and *Trifolium* should absorb more P by AMF hyphae than by its coarse root system (Sprent and James, 2007; Saia et al., 2014; Unger et al., 2016), while *Lolium* should be less colonized and rely more on root P uptake (Reinhart et al., 2012; Friede et al., 2016; Unger et al., 2016).

Regarding the control treatment, results indicate a substantial increase in the biomass for both *Lolium* and *Trifolium* in the presence of AMF; thus both species greatly benefited from P uptake through mycorrhiza. This was surprising for the facultative mycotroph *Lolium*, but was confirmed by high colonization rates of the grass. Indeed, *L. multiflorum* has been characterized as a species always associated with arbuscular mycorrhiza (Wang and Qiu, 2006; Hempel et al., 2013). Biomass in the mycorrhizal state was affected by competition with a general, but often non-significant, trend of decreasing biomass in both species with increasing *Lolium* count in the mixture, which is indicative of *Lolium* being the stronger competitor of the two species. Indeed, RNE-P values showed a strong competitive effect on *Trifolium*, which increased with *Lolium* count, while *Lolium*-P acquisition was facilitated by higher *Trifolium* numbers in the mixture. Similarly, the MPD of both species increased with increasing counts of *Trifolium* in the mixture, indicating larger mycorrhizal benefits for P acquisition with clover plants sustaining the AMF. However, neither effect was significant in any of the species. An explanation for the trend toward higher competitive ability of *Lolium* as compared to *Trifolium* may be found in the colonization data, with a high degree of mycorrhizal colonization often being associated with a large phosphorus uptake (Smith and Smith, 2011; Höpfner et al., 2014; Unger et al., 2016). Highest mycorrhizal colonization of *Lolium* in the control treatment was found in pots with one *Lolium* individual and three *Trifolium* individuals, indicating a trend toward higher P-uptake potential of the grass in the presence of highly colonized clover roots. High infectiveness of a large mycorrhizal network provided by another species, leading to high colonization rates in the species usually less colonized when grown without neighbors, has also been observed in other studies (Höpfner et al., 2015; Friede et al., 2016). Further, competition in the mycorrhizal network may be mediated by the fungus, supplying the host that has a higher leaf biomass and C-supply (in our case *L. multiflorum*) with relatively more nutrients (Fellbaum et al., 2014). In addition, the ratio of arbuscules to vesicles found in *Lolium* roots was highly dependent on species composition in the mixture, with the grass, in the presence of *Trifolium*, being able to build more arbuscules, which form the interface for nutrient exchange (Smith and Read, 2008), in comparison to vesicles, which imply increased carbon drain by the fungus (Smith and Read, 2008; Sendek et al., 2019). The modulation of investment into P-delivering vs. carbon-draining structures in *Lolium* through *Trifolium* may thus, partly be responsible for the observed facilitation of the grass by the clover.

### Effects of Fertilization on Arbuscular Mycorrhizal Fungi Promotion of Competitive Outcome Between *Trifolium* and *Lolium*

We hypothesized that the effect of AMF on the competitive balance between *Lolium* and *Trifolium* would be affected by N- and P-fertilization (Munoz and Weaver, 1999; Friede et al., 2016). N-fertilization was expected to increase photosynthetic capacity (Evans, 1989) in both plant species and thus increase carbon input into the fungal network, resulting in higher colonization, particularly in the highly mycotrophic legume (Unger et al., 2016), leading to a higher C–P trade benefit in the grass than in the clover. In addition, the presumed advantage of the rhizobial symbiosis of *Trifolium* would be diminished under these conditions (Reinprecht et al., 2020). P-fertilization was expected to increase the competitive ability of the legume over the grass (Ren et al., 2017; Mitran et al., 2018). In addition, a shift in the mutualistic relationship of the fungus with the plant toward a detrimental situation for both plant species (Johnson, 2010) was expected, causing the mycorrhizal grass to show smaller or even negative mycorrhizal growth responses in comparison to the clover (Friede et al., 2016).

In relation to N-fertilization, previous studies found a strong competitive shift toward higher grass performance with increasing levels of fertilizer N in mixtures with clover (Munoz and Weaver, 1999; Silvertown et al., 2006; Song et al., 2011; Wagg et al., 2011; Hacker et al., 2015; Grygierzec et al., 2020). However, in our experiment, N-fertilization surprisingly did not show an effect on biomass in either plant species, suggesting N not being a limiting factor and additional N not adding any growth advantages. Thus, the competitive balance between the grass and the legume did not show the expected change with N-addition under both mycorrhizal and non-mycorrhizal conditions, which is in line with RNE and RNE-P being similar to the control treatment in both species.

Slightly lower colonization in *Lolium* in the +N treatment may explain the trend toward smaller tissue P concentration and MPD in this species (Smith and Smith, 2011; Höpfner et al., 2014; Unger et al., 2016). Also, the A:V ratio was smaller than under control conditions, suggesting higher C-input into the mycorrhizal network by *Lolium*. Thus, the C-P trade benefit for *Lolium* was probably less favorable with N addition as compared to the control treatment, while this was not observed in *Trifolium*. Nevertheless, this was of minor importance for the competitive outcome, as growth and competition effects were not different from the control treatment (see Section “Effects of Arbuscular Mycorrhizal Fungi on competitive balance between *Trifolium* and *Lolium*”).

Phosphorous-fertilization on the other hand, had a positive growth effect on non-mycorrhizal *Lolium* and mycorrhizal individuals of both species, this effect being more pronounced in the grass, which reached nearly double the biomass than in the other treatments.

In the non-mycorrhizal state, the positive growth response of *Lolium* with additional P, however, did not affect the competition with *Trifolium* in the non-mycorrhizal state. This was mainly caused by the *Trifolium* plants not being able to accumulate enough phosphorus to develop a biomass increase in the absence of mycorrhiza (e.g., Janos, 2007; Smith and Read, 2008) and we can assume that in this state, competition for nutrients in both species did not play a major role, which is also supported by tissue nutrients not being markedly affected by the applied competition treatments, with RNE-P values being relatively small across all treatments.

When mycorrhizal, there were no obvious changes in MGD or MPD in *Trifolium* in the +P treatment, while in *Lolium*, both parameters tended to decrease and at times even being negative, as compared to control plants, which might be explained by a smaller mutualistic fungus–plant relationship under P-fertilization (Johnson, 2010; Friede et al., 2016). This, however, did not result in the expected change in the competitive ability in the grass, which achieved markedly, though non-significantly, higher biomass with increasing clover count. An explanation might be found again in the adjustment of the C–P trade benefit of the grass. First, under +P conditions, *Lolium* lowered colonization levels, with the direct root uptake pathway becoming increasingly important (Smith et al., 2009; Smith and Smith, 2011). Second, with clover present in the mixture, the grass tended to adjust the ratio of vesicles to arbuscules toward less C-investment in the mycorrhizal network and more fungal P-exchange interface. *Trifolium* on the other hand was significantly smaller when there were more *Lolium* plants in the mixture, which resulted in relatively higher RNE values in this species with P-fertilization. However, under P-fertilization, RNE-P in *Trifolium* was smaller as compared to the other treatments, indicating that competition for P with the grass was not the reason for the lower performance of *Trifolium* in the presence of *Lolium*, but more likely, a higher carbon allocation to the mycelial network. Indeed, in the +P treatment, A:V ratios in *Lolium* increased, while *Trifolium* exhibited a decreased ratio. Although, there is evidence that AMF symbiosis is stabilized by physiological mechanisms, bidirectionally controlling the reciprocal exchange of nutrients (Kiers et al., 2011), differential C-P trade benefits (van der Heijden and Horton, 2009; Höpfner et al., 2015; Friede et al., 2016) in mycelial networks may be a driver of competitive outcome.

## Conclusion and Outlook

In this study, we addressed mycorrhizal effects on the competitive ability of the prominent grass (i.e., *L. multiflorum*) and the prominent legume (i.e., *T. subterraneum*) in SBP with different N- and P-fertilization. Although the effects of competition on the studied parameters were generally weak, we showed clear and interesting trends of species composition in the pots on biomass, plant nutrient concentrations, and mycorrhizal investment.

From our results, we may conclude that in this study, *Lolium* was clearly a better competitor for phosphorus than *Trifolium* and, in contrast to our hypothesis, was facilitated through the presence of mycorrhiza provided and sustained by the clover. Mycorrhizal *Trifolium* on the other hand, suffered from P-competition by the grass, showing a lower performance with increasing number of *Lolium* individuals in the pot. While N-fertilization in the mycorrhizal treatments did not change the competitive balance between mycorrhizal grass and clover, P-fertilization had a marked effect on both species with stronger biomass increases in *Lolium* as compared to *Trifolium*. Although the grass showed smaller mycorrhizal benefits, while clover did not, *Lolium* obtained a competitive advantage through mycorrhiza with extra P. This may be explained by the facultative mycotrophic *Lolium* being able to control the C–P trade of the AMF symbiosis by better adjustment of the A:V ratio than the highly responsive clover. The more plastic usage of the direct root uptake pathway over the mycorrhizal pathway for P-acquisition in *Lolium* than in *Trifolium*, and thereby ensuring a beneficial C–P trade in the common mycelial network, may thus be a key factor for the competitive success of the grass.

What are the implications of our results for the *in situ* SBP?

First, our results indicate that AMF were clearly beneficial for both species. As the majority of herbaceous plant species have AMF symbionts (Wang and Qiu, 2006), and since AMF abundance tends to be much higher in grasslands than in other ecosystems (Treseder and Cross, 2006), mycorrhizal abundance in SBP, *in situ*, can be expected to be high, good management practices that encourage soil conditions (Guzman et al., 2021) and grazing management (Cavagnaro et al., 2019) favorable for AMF abundance will be crucial for the success of the SBP practice. In addition, our results clearly indicate that P-fertilization is necessary for the good performance of the SBP mixture.

Second, our results showed that competition between the two species was influenced by the available soil P with mycorrhizal *Trifolium* exhibiting lower biomass with higher *Lolium* count in the mixture and vice versa. As success and sustainability of SBP are dependent on a good legume performance, a higher legume: grass ratio in the seed mixtures for SBP is therefore advisable.

Extrapolation of our results to *in situ* conditions is not totally straightforward. We used pre-inoculated plants, which does not reflect natural field conditions, where seedlings are usually inoculated *via* life hyphae from mycorrhizal networks already established by adult plants. However, in the studied Mediterranean system, this may not matter as much, as here, adult plants and life hyphae do not exist in large amounts after the summer drought and the system reestablishes from seedlings being inoculated by spores. In addition, in our study, the substrate only contained a single AMF isolate while *in situ* soils contain a large diversity of bacteria and fungi, and micro- and macrofauna, with the functional complementarity among species within an AMF community colonizing a single root system undoubtedly affecting mycorrhizal responsiveness (Jansa et al., 2008). Nevertheless, several studies have reported on equal benefits to the host of single-species AMF inoculum as compared to a mixed inoculum treatment (Boyer et al., 2015; Armada et al., 2016).

Further, our study neither aims to assess the significance of rhizobial symbiosis, nor the effects of different fertilizer levels on this symbiosis, even though it is important for N-uptake in legumes (Saia et al., 2014). Different degrees of root nodulation due to direct effects of the mycorrhizal and nutrient treatments on root system size may have affected the accumulation of biomass and the N-status of the legume. However, as nodules were observed in *Trifolium* across all treatments and N-status of neither species depended on competition or fertilization, effects of nitrogen acquisition through rhizobial symbiosis probably played a minor role for the outcome of the experiment.

Notwithstanding the limitations in the transferability of results from a pot study to the *in situ* situation, the main findings of this study are important for the SBP practice.

## Data Availability Statement

The raw data supporting the conclusions of this article will be made available by the authors, without undue reservation.

## Author Contributions

SU, FMH, and KS designed the experiment. SU and MJ accomplished the data analysis, data interpretation and presentation, and wrote the manuscript. All authors contributed to the article and approved the submitted version.

## Conflict of Interest

The authors declare that the research was conducted in the absence of any commercial or financial relationships that could be construed as a potential conflict of interest. The reviewer AN declared a shared affiliation with one of the authors MJ to the handling editor at the time of the review.

## Publisher’s Note

All claims expressed in this article are solely those of the authors and do not necessarily represent those of their affiliated organizations, or those of the publisher, the editors and the reviewers. Any product that may be evaluated in this article, or claim that may be made by its manufacturer, is not guaranteed or endorsed by the publisher.

## References

[B1] Armada E. López-Castillo O. Roldán A. Azcón R. (2016). Potential of mycorrhizal inocula to improve growth, nutrition and enzymatic activities in *Retama sphaerocarpa* compared with chemical fertilization under drought conditions. *J. Soil Sci. Plant Nutr.* 16 380–399. 10.4067/S0718-95162016005000035 27315006

[B2] Ashgari H. R. Cavagnaro T. R. (2012). Arbuscular mycorrhizas reduce nitrogen loss *via* leaching. *PLoS One* 7:e29825. 10.1371/journal.pone.0029825 22253790 PMC3254610

[B3] Beringer J. E. Brewin N. Johnston A. W. Schulman H. M. Hopwood D. A. (1979). The Rhizobium-legume symbiosis. *Proc. R. Soc. Lond. B Biol. Sci.* 204 219–233. 10.1098/rspb.1979.0024 36624

[B4] Boyer L. R. Brain P. Xu X. M. Jeffries P. (2015). Inoculation of drought-stressed strawberry with a mixed inoculum of two arbuscular mycorrhizal fungi: effects on population dynamics of fungal species in roots and consequential plant tolerance to water deficiency. *Mycorrhiza* 25 215–227. 10.1007/s00572-014-0603-6 25186649

[B5] Callaway R. M. Brooker R. W. Choler P. Kikvidze Z. Lortie C. J. Michalet R. (2002). Positive interactions among alpine plants increase with stress. *Nature* 417 844–848. 10.1038/nature00812 12075350

[B6] Carneiro J. P. Simões N. Maçãs I. D. Tavares de Sousa M. M. (2008). Pasture improvement in montado extensive farming systems. *Opt. Med. A* 79 193–197.

[B7] Cavagnaro R. A. Pero E. Dudinszky N. Golluscio R. A. Grimoldi A. A. (2019). Under pressure from above: overgrazing decreases mycorrhizal colonization of both preferred and unpreferred grasses in the Patagonian steppe. *Fungal Ecol.* 40 92–97. 10.1016/j.funeco.2018.09.003

[B8] Crush J. R. (1995). Effect of VA mycorrhizas on phosphorus uptake and growth of white clover (*Trifolium repens* L.) growing in association with ryegrass (*Lolium perenne* L.). *N Z. J. Agric. Res.* 38 303–306. 10.1080/00288233.1995.9513131

[B9] Daisog H. Sbrana C. Cristani C. Moonen A.-C. Giovannetti M. Bàrberi P. (2012). Arbuscular mycorrhizal fungi shift competitive relationships among crop and weed species. *Plant Soil* 353 395–408. 10.1007/s11104-011-1040-3

[B10] Evans J. R. (1989). Photosynthesis and nitrogen relationships in leaves of C3 plants. *Oecologia* 78 8–19. 10.1007/BF00377192 28311896

[B11] Fellbaum C. R. Gachomo E. W. Beesetty Y. Choudhari S. Strahan G. D. Pfeffer P. E. (2012). Carbon availability triggers fungal nitrogen uptake and transport in arbuscular mycorrhizal symbiosis. *Proc. Natl. Acad. Sci. U S A.* 109 2666–2671. 10.1073/pnas.1118650109 22308426 PMC3289346

[B12] Fellbaum C. R. Mensah J. A. Cloos A. J. Strahan G. E. Pfeffer P. E. Kiers E. T. (2014). Fungal nutrient allocation in common mycorrhizal networks is regulated by the carbon source strength of individual host plants. *New Phytol.* 203 646–656. 10.1111/nph.12827 24787049

[B13] Friede M. Unger S. Hellmann C. Beyschlag W. (2016). Conditions promoting mycorrhizal parasitism are of minor importance for competitive interactions in two differentially mycotrophic species. *Front. Plant Sci.* 7:1465. 10.3389/fpls.2016.01465 27729924 PMC5037182

[B14] Friede M. Unger S. Heuer L. Stammes R. Beyschlag W. (2017). Nitrogen limitation impairs plant control over the arbuscular mycorrhizal symbiosis in response to phosphorus and shading in two European sand dune species. *Plant Ecol.* 219 17–29. 10.1007/s11258-017-0774-2

[B15] Grygierzec B. Musiał K. Luty L. (2020). Sowing ratio, NS fertilisation and interactions of *Lolium* sp. and *Festulolium* grown in mixtures with *Trifolium repens*. *Plant Soil Environ.* 66 395–402. 10.17221/82/2020-PSE

[B16] Guzman A. Montes M. Hutchins L. DeLaCerda G. Yang P. Kakouridis A. (2021). Crop diversity enriches arbuscular mycorrhizal fungal communities in an intensive agricultural landscape. *New Phytol.* 231 447–459. 10.1111/nph.17306 33638170 PMC9292320

[B17] Hacker N. Ebeling A. Gessler A. Gleixner G. González Macé O. de Kroon H. (2015). Plant diversity shapes microbe-rhizosphere effects on P mobilisation from organic matter in soil. *Ecol. Lett.* 18 1356–1365. 10.1111/ele.12530 26415778

[B18] Hempel S. Götzenberger L. Kühn I. Michalski S. G. Rillig M. C. Zobel M. (2013). Mycorrhizas in the Central European flora: relationships with plant life history traits and ecology. *Ecology* 94 1389–1399. 10.1890/12-1700.1 23923502

[B19] Hernández-Esteban A. López-Díaz M. L. Cáceres Y. Moreno G. (2019). Are sown legume-rich pastures effective allies for the profitability and sustainability of Mediterranean dehesas? *Agroforest. Syst.* 93 2047–2065. 10.1007/s10457-018-0307-6

[B20] Hetrick B. A. D. Hartnett D. C. Wilson G. W. Gibson D. J. (1994). Effects of mycorrhizae, phosphorus availability, and plant density on yield-relationships among competing tallgrass prairie grasses. *Can. J. Bot.* 72 168–176. 10.1139/b94-023

[B21] Hill J. O. Simpson R. J. Wood J. T. Moore A. D. Chapman D. F. (2005). The phosphorus and nitrogen requirements of temperate pasture species and their influence on grassland botanical composition. *Aust. J. Agric. Res.* 56 1027–1039. 10.1071/AR04279

[B22] Hoagland D. R. Arnon D. I. (1950). The water-culture method for growing plants without soil. *Calif. Agric. Exp. Stn. Circ.* 347 1–32.

[B23] Hodge A. (2003). Plant nitrogen capture from organic matter as affected by spatial dispersion, interspecific competition and mycorrhizal colonization. *New Phytol.* 157 303–314. 10.1046/j.1469-8137.2003.00662.x 33873633

[B24] Hodge A. Fitter A. H. (2010). Substantial nitrogen acquisition by arbuscular mycorrhizal fungi from organic material has implications for N cycling. *Proc. Natl. Acad. Sci. U S A.* 107 13754–13759. 10.1073/pnas.1005874107 20631302 PMC2922220

[B25] Hodge A. Helgason T. Fitter T. H. (2010). Nutritional ecology of arbuscular mycorrhizal fungi. *Fungal Ecol.* 3 267–273. 10.1016/j.funeco.2010.02.002

[B26] Höpfner I. Beyschlag W. Bartelheimer M. Werner C. Unger S. (2015). Role of mycorrhization and nutrient availability in competitive interactions between the grassland species *Plantago lanceolata* and *Hieracium pilosella*. *Plant Ecol.* 216 887–899. 10.1007/s11258-015-0476-6

[B27] Höpfner I. Friede M. Unger S. Beyschlag W. (2014). Potential advantages of highly mycotrophic foraging for the establishment of early successional pioneer plants on sand. *Funct. Plant Biol.* 42 95–104. 10.1071/FP14097 32480656

[B28] Janos D. P. (2007). Plant responsiveness to mycorrhizas differs from dependence upon mycorrhizas. *Mycorrhiza* 17 75–91. 10.1007/s00572-006-0094-1 17216499

[B29] Jansa J. Smith F. A. Smith F. E. (2008). Are there benefits of simultaneous colonization by different arbuscular mycorrhizal fungi? *New Phytol.* 177 779–789. 10.1111/j.1469-8137.2007.02294.x 18042204

[B30] Johnson N. C. (2010). Resource stoichiometry elucidates the structure and function of arbuscular mycorrhizas across scales. *New Phytol.* 185 631–647. 10.1111/j.1469-8137.2009.03110.x 19968797

[B31] Jongen M. Förster A. C. Unger S. (2019). Overwhelming effects of autumn-time drought during seedling establishment impair recovery potential in sown and semi-natural pastures in Portugal. *Plant Ecol.* 220 183–197. 10.1007/s11258-018-0869-4

[B32] Jongen M. Unger S. Fangueiro D. Cerasoli S. Silva J. M. N. Pereira J. S. (2013). Resilience of montado understorey to experimental precipitation variability fails under severe natural drought. *Agr. Ecosyst. Environ.* 178 18–30. 10.1016/j.agee.2013.06.014

[B33] Kiers E. T. Duhamel M. Beesetty Y. Mensah J. A. Franken O. Verbruggen E. (2011). Reciprocal rewards stabilize cooperation in the mycorrhizal symbiosis. *Science* 333 880–883. 10.1126/science.1208473 21836016

[B34] Knapp A. K. Ciais P. Smith M. D. (2017). Reconciling inconsistencies in precipitation–productivity relationships: implications for climate change. *New Phytol.* 214 41–47. 10.1111/nph.14381 28001290

[B35] Koide R. T. Li M. G. (1989). Appropriate controls for vesicular-arbuscular mycorrhiza research. *New Phytol.* 111 35–44. 10.1111/j.1469-8137.1989.tb04215.x

[B36] Koide R. T. Goff M. D. Dickie I. A. (2000). Component growth efficiencies of mycorrhizal and nonmycorrhizal plants. *New Phytol.* 148 163–168. 33863038 10.1046/j.1469-8137.2000.00741.x

[B37] Markham J. H. Chanway C. P. (1996). Measuring plant neighbour effects. *Funct. Ecol.* 10 548–549.

[B38] McCaskill M. R. Mitchell M. L. Zollinger R. Armstrong R. D. Partington D. (2019). Dry matter and nutritive value responses of native, naturalised and sown pasture species to soil Olsen P. *Crop Pasture Sci.* 70 1097–1109. 10.1071/CP18544

[B39] McGonigle T. Miller M. H. Evans D. G. Fairchild G. L. Swan J. A. (1990). A new method which gives an objective-measure of colonization of roots by vesicular arbuscular mycorrhizal fungi. *New Phytol.* 115 495–501. 10.1111/j.1469-8137.1990.tb00476.x 33874272

[B40] Merrild M. P. Ambus P. Rosendahl S. Jakobsen I. (2013). Common arbuscular mycorrhizal networks amplify competition for phosphorus between seedlings and established plants. *New Phytol.* 200 229–240. 10.1111/nph.12351 23738787

[B41] Mitran T. Meena R. S. Lal R. Layek J. Kumar S. Datta R. (2018). “Role of soil phosphorus on legume production,” in *Legumes for Soil Health and Sustainable Management*, eds Meena R. Das A. Yadav G. Lal R. (Singapore: Springer), 487–510. 10.1007/978-981-13-0253-4_15

[B42] Montesinos-Navarro A. Valiente-Banuet A. Verdú M. (2019). Processes underlying the effect of mycorrhizal symbiosis on plant-plant interactions. *Fungal Ecol.* 40 98–106. 10.1016/j.funeco.2018.05.003

[B43] Munoz A. E. Weaver R. W. (1999). Competition between subterranean clover and rygrass for uptake of 15N-labeled fertilizer. *Plant Soil.* 211 173–178. 10.1023/A:1004646319700

[B44] Reinhart K. O. Wilson G. W. T. Rinella M. J. (2012). Predicting plant responses to mycorrhizae: integrating evolutionary history and plant traits. *Ecol. Lett.* 15 689–695. 10.1111/j.1461-0248.2012.01786.x 22507627

[B45] Reinprecht Y. Schram L. Marsolais F. Smith T. H. Hill B. Pauls K. P. (2020). Effects of nitrogen application on nitrogen fixation in common bean production. *Front. Plant Sci.* 11:1172. 10.3389/fpls.2020.01172 32849727 PMC7424037

[B46] Ren F. Song W. Chen L. Mi Z. Zhang Z. Zhu W. (2017). Phosphorus does not alleviate the negative effect of nitrogen enrichment on legume performance in an alpine grassland. *J. Plant Ecol.* 10 822–830. 10.1093/jpe/rtw089

[B47] Saia S. Amato G. Frenda A. S. Giambalvo D. Ruisi P. (2014). Influence of arbuscular mycorrhizae on biomass production and nitrogen fixation of berseem clover plants subjected to water stress. *PLoS One* 9:e90738. 10.1371/journal.pone.0090738 24595111 PMC3940947

[B48] Sales-Baptista E. d’Abreu M. C. Ferraz-de-Oliveira M. I. (2016). Overgrazing in the montado? The need for monitoring grazing pressure at paddock scale. *Agrofor. Syst.* 90 57–68. 10.1007/s10457-014-9785-3

[B49] Scheublin T. R. van Logtestijn R. S. P. van der Heijden M. G. A. (2007). Presence and identity of arbuscular mycorrhizal fungi influence competitive interactions between plant species. *J. Ecol.* 95 631–638. 10.1111/j.1365-2745.2007.01244.x

[B50] Schüller H. (1969). Die CAL-Methode, eine neue Methode zur Bestimmung des pflanzenverfügbaren Phosphates in Böden. *Z. Pflanzenernähr Bodenkd.* 123 48–63. 10.1002/jpln.19691230106

[B51] Sendek A. Karakoç C. Wagg C. Domínguez-Begines J. Martucci, do Couto G. (2019). Drought modulates interactions between arbuscular mycorrhizal fungal diversity and barley genotype diversity. *Sci. Rep.* 9:9650. 10.1038/s41598-019-45702-1 31273222 PMC6609766

[B52] Silvertown J. Poulton P. Johnston E. Edwards G. Heard M. Biss P. M. (2006). The Park Grass Experiment 1856–2006: Its contribution to ecology. *J. Ecol.* 94 801–814. 10.1111/j.1365-2745.2006.01145.x

[B53] Smith F. A. Smith S. E. (2013). How useful is the mutualism-parasitism continuum of arbuscular mycorrhizal functioning? *Plant Soil.* 363 7–18. 10.1007/s11104-012-1583-y

[B54] Smith F. A. Grace E. J. Smith S. E. (2009). More than a carbon economy: nutrient trade and ecological sustainability in facultative arbuscular mycorrhizal symbioses. *New Phytol.* 182 347–358. 10.1111/j.1469-8137.2008.02753.x 19207688

[B55] Smith M. D. Hartnett D. C. Wilson G. W. T. (1999). Interacting influence of mycorrhizal symbiosis and competition on plant diversity in tallgrass prairie. *Oecologia* 121 574–582. 10.1007/s004420050964 28308367

[B56] Smith S. E. Read D. J. (2008). *Mycorrhizal symbiosis.* London: Academic Press.

[B57] Smith S. E. Smith F. A. (2011). Roles of arbuscular mycorrhizas in plant nutrition and growth: new paradigms from cellular to ecosystem scales. *Annu. Rev. Plant Biol.* 62 227–250. 10.1146/annurev-arplant-042110-103846 21391813

[B58] Smith S. E. Jakobsen I. Grønlund M. Smith F. A. (2011). Roles of arbuscular mycorrhizas in plant phosphorus (P) nutrition: interactions between pathways of P uptake in arbuscular mycorrhizal (AM) roots have important implications for understanding and manipulating P acquisition. *Plant Physiol.* 156 1050–1057. 10.1104/pp.111.174581 21467213 PMC3135927

[B59] Smith S. E. Smith F. A. Jakobsen I. (2003). Mycorrhizal fungi can dominate phosphate supply to plants irrespective of growth responses. *Plant Physiol.* 133 16–20. 10.1104/pp.103.024380 12970469 PMC1540331

[B60] Song L. Bao X. Liu X. Zhang Y. Christie P. Fangmeier A. (2011). Nitrogen enrichment enhances the dominance of grasses over forbs in a temperate steppe ecosystem. *Biogeosciences* 8 2341–2350. 10.5194/bg-8-2341-2011

[B61] Sprent J. I. James E. K. (2007). Legume evolution: where do nodules and mycorrhizas fit in? *Plant Physiol.* 144 575–581. 10.1104/pp.107.096156 17556520 PMC1914177

[B62] Teixeira R. F. M. Proença V. Crespo D. Valada T. Domingos T. (2015). A conceptual framework for the analysis of engineered biodiverse pastures. *Ecol. Eng.* 77 85–97. 10.1016/j.ecoleng.2015.01.002

[B63] Treseder K. Cross A. (2006). Global distributions of arbuscular mycorrhizal fungi. *Ecosystems* 9 305–316.

[B64] Unger S. Jongen M. (2015). “Consequences of changing precipitation patterns for productivity and diversity in grassland ecosystems: a review,” in *Progress in Botany*, Vol. 76 eds Lüttge U. Beyschlag W. (Heidelberg: Springer), 347–393. 10.1007/978-3-319-08807-5_14

[B65] Unger S. Friede M. Hundacker J. Volkmar K. Beyschlag W. (2016). Allocation trade-off between root and mycorrhizal surface defines nitrogen and phosphorus relations in 13 grassland species. *Plant Soil* 407 279–292. 10.1007/s11104-016-2994-y

[B66] van der Heijden M. G. A. Horton T. R. (2009). Socialism in soil? The importance of mycorrhizal fungal networks for facilitation in natural ecosystems. *J. Ecol.* 97 1139–1150. 10.1111/j.1365-2745.2009.01570.x

[B67] van Oijen M. Bellocchi G. Höglind M. (2018). Effects of climate change on grassland biodiversity and productivity: the need for a diversity of models. *Agronomy* 8:14. 10.3390/agronomy8020014

[B68] Vierheilig H. Coughlan A. P. Wyss U. Piché Y. (1998). Ink and vinegar, a simple staining technique for arbuscular-mycorrhizal fungi. *Appl. Environ. Microbiol.* 64 5004–5007. 10.1128/AEM.64.12.5004-5007.1998 9835596 PMC90956

[B69] Wagg C. Jansa J. Stadler M. Schmid B. van der Heijden M. G. A. (2011). Mycorrhizal fungal identity and diversity relaxes plant–plant competition. *Ecology* 92 1303–1313. 10.1890/10-1915.1 21797158

[B70] Wang B. Qiu Y. L. (2006). Phylogenetic distribution and evolution of mycorrhizas in land plants. *Mycorrhiza* 16 299–363. 10.1007/s00572-005-0033-6 16845554

[B71] Watanabe F. S. Olsen S. R. (1965). Test of an ascorbic acid method for determining phosphorus in water and NaHCO_3_ extracts from soils. *Soil Sci. Soc. Am. Pro.* 29 677–678. 10.2136/sssaj1965.03615995002900060025x

[B72] White R. P. Murray S. Rohweder M. (2000). *Pilot analysis of global ecosystems: grassland ecosystems.* Washington, DC: World Resources Institute.

[B73] Whiteside M. D. Digman M. A. Gratton E. Treseder K. K. (2012). Organic nitrogen uptake by arbuscular mycorrhizal fungi in a boreal forest. *Soil Biol. Biochem.* 55 7–13. 10.1016/j.soilbio.2012.06.001 24371363 PMC3871874

