# Supplementary material for: Arbuscular Mycorrhizal Fungi and Nutrition Determine the Outcome of Competition Between Lolium multiflorum and Trifolium subterraneum
Source: Front Plant Sci. 2021 Dec 23;12:778861. doi: 10.3389/fpls.2021.778861 (PMC8733683; doi:10.3389/fpls.2021.778861)
Supplement: Supplementary file 1 [file Data_Sheet_1.pdf]

Supplementary Material

Supplementary Figures

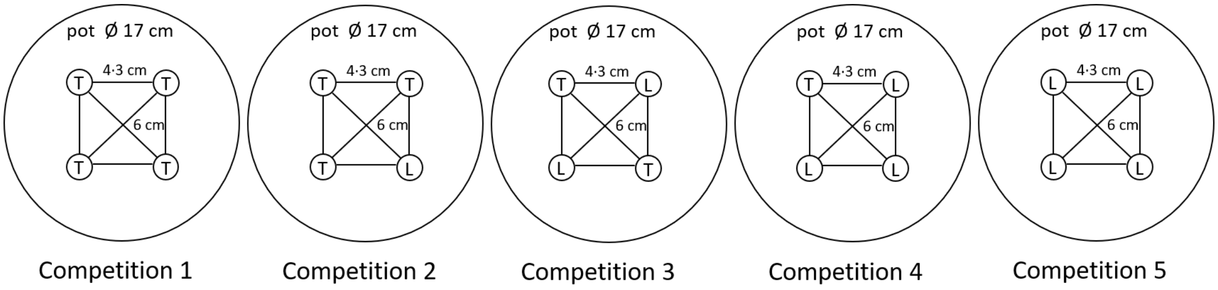

Figure S1: Planting design and competition levels, with T = *Trifolium subterraneum* and L = *Lolium multiflorum*. Neighboring plants had a distance of approximately 4.3 cm and diagonally arrange plants a distance of approximately 6 cm.

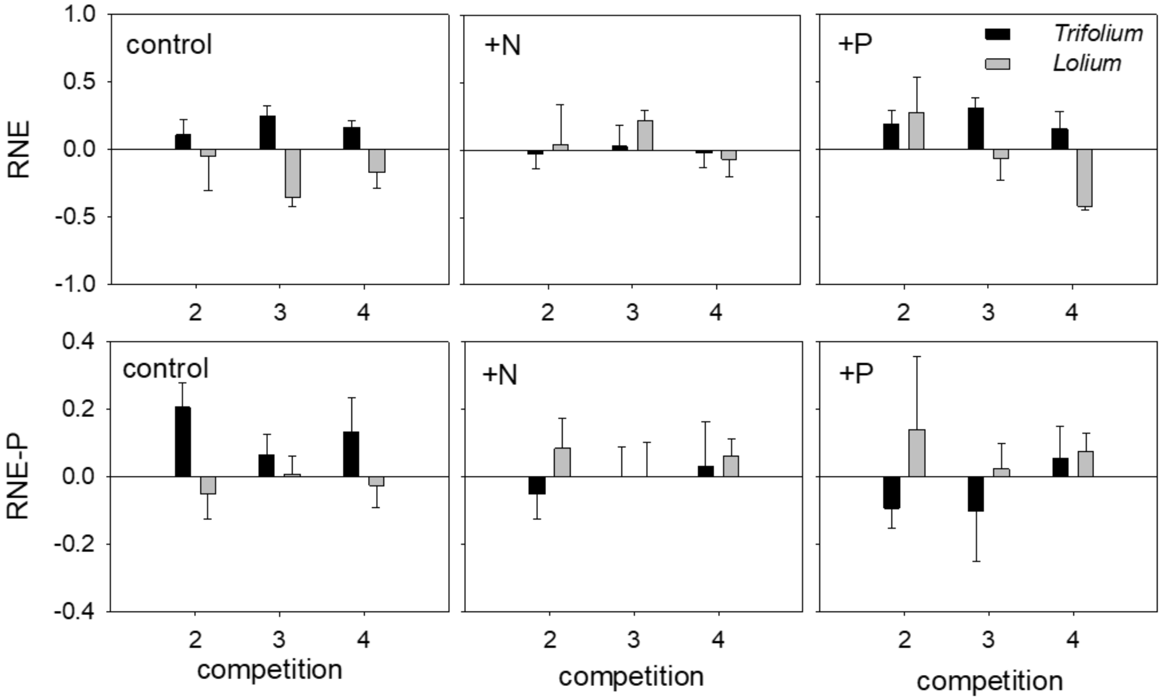

Figure S2: Relative neighbor effect (RNE) and relative neighbor effect for P-competition (RNE-P) of non-mycorrhizal (NM) *Trifolium subterraneum* (■) and *Lolium multiflorum* (■), subjected to standard fertilization (control), enhanced N fertilizer (+N) or enhanced P fertilizer (+P), in the competition treatments 2, 3 and 4. Data represent mean ± standard error, n = 5.

Supplementary Tables

Table S1: Composition of the modified Hoagland nutrient solution (Hoagland and Arnon 1950) for the control (standard fertilizer), enhanced phosphate content (+P fertilizer), or the enhanced nitrogen content (+N fertilizer). Stock solutions were 1 M. Micronutrients and their stock solutions were H_3BO_3 (25 µM), $MnSO_4$ (2 µM), $ZnSO_4$ (2 µM), $CuSO_4$ (0.5 µM), MoO_3 (0.5 µM).

**compound**	**volume of stock solution (ml) per liter for:**		
	standard fertilizer	+P fertilizer	+N fertilizer
KNO_3	6	5	10
$Ca(NO_3)_2 \cdot 4H_2O$	2	2.5	2
$(NH_4)_2SO_4$	1	1	1
KH_2PO_4	0	3	0
$(NH_4)_2HPO_4$	1	1	1
$MgSO_4 \cdot 7H_2O$	2	2	2
KCl	6	4	2
$CaCl_2$	0.5	0	0.5
NaCl	1.5	2	1.5
Na_2SO_4	2	2	2
Fe-Citrat ($FeC_6H_5O_7$)	1	1	1
Micronutrients	1	1	1

Table S2: Results of three-way ANOVA for factors competition (1-5), mycorrhization (NM, AM), and fertilization (control, +N, +P) on soil inorganic P and N. Data stated are P-values, with significant effects (P < 0.05) given in bold. Different letters for the factors competition, and fertilization indicate significantly different means (Tukey HSD post-hoc, P < 0.05).

	Soil P (mg kg^{-1})	Soil N (mg kg^{-1})
Competition (C) (1,2,3,4,5)	0.61	**0.003** (a,ab,ab,b,b)
Mycorrhization (M)	**<0.001**	0.53
Fertilization (F) (control,+N,+P)	**<0.001** (b,b,a)	0.83
C × M	0.39	0.40
C × F	0.88	0.95
M × F	0.97	0.95
C × M × F	0.92	0.83

Table S3: Soil P (mg kg^{-1}) in the non-mycorrhizal and mycorrhizal pots with standard fertilization (control), enhanced N fertilizer (+N) or enhanced P fertilizer (+P). As factor competiton is not significant, values for the five competition treatmens are pooled. Data represents mean ± standard error, n = 25. Different letters indicate significantly different means (Tukey HSD post-hoc, P < 0.05).

Mycorrhization / Fertilization	Soil P (mg kg^{-1})
NM, control	5.11±0.380 b
NM, +N	5.72±0.458 b
NM, +P	7.83±0.604 a
AM, control	3.33±0.189 c
AM, +N	3.61±0.229 c
AM, +P	5.38±0.433 b

Table S4: Soil N (mg kg^{-1}) in the five competition treatments. As factors mycorrhization and fertilization are not significant, average values are given for each of the five competition treatments. Data represents mean ± standard error, n = 30. Different letters indicate significantly different means (Tukey HSD post-hoc, P < 0.05).

Competition	Soil N (mg kg^{-1})
1	44.15±4.244 a
2	32.22±3.387 ab
3	30.07±4.289 ab
4	26.66±3.098 b
5	24.85±2.295 b
