# Supplementary figures and images for: Arbuscular Mycorrhizal Fungi and Nutrition Determine the Outcome of Competition Between Lolium multiflorum and Trifolium subterraneum
Source: Front Plant Sci. 2021 Dec 23;12:778861. doi: 10.3389/fpls.2021.778861 (PMC8733683; doi:10.3389/fpls.2021.778861)

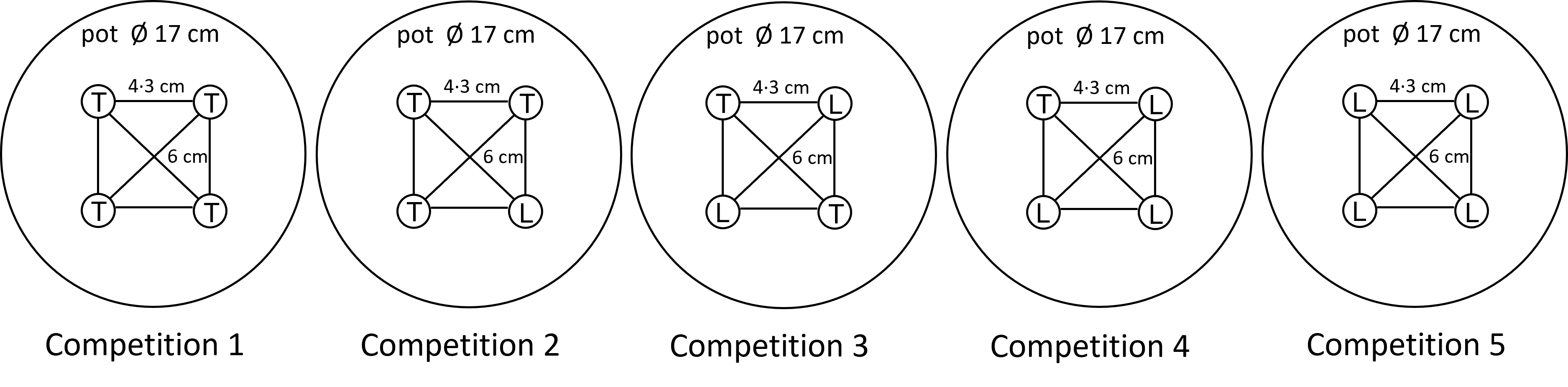

Supplement: Supplementary file 2 [file Image_1.TIF]

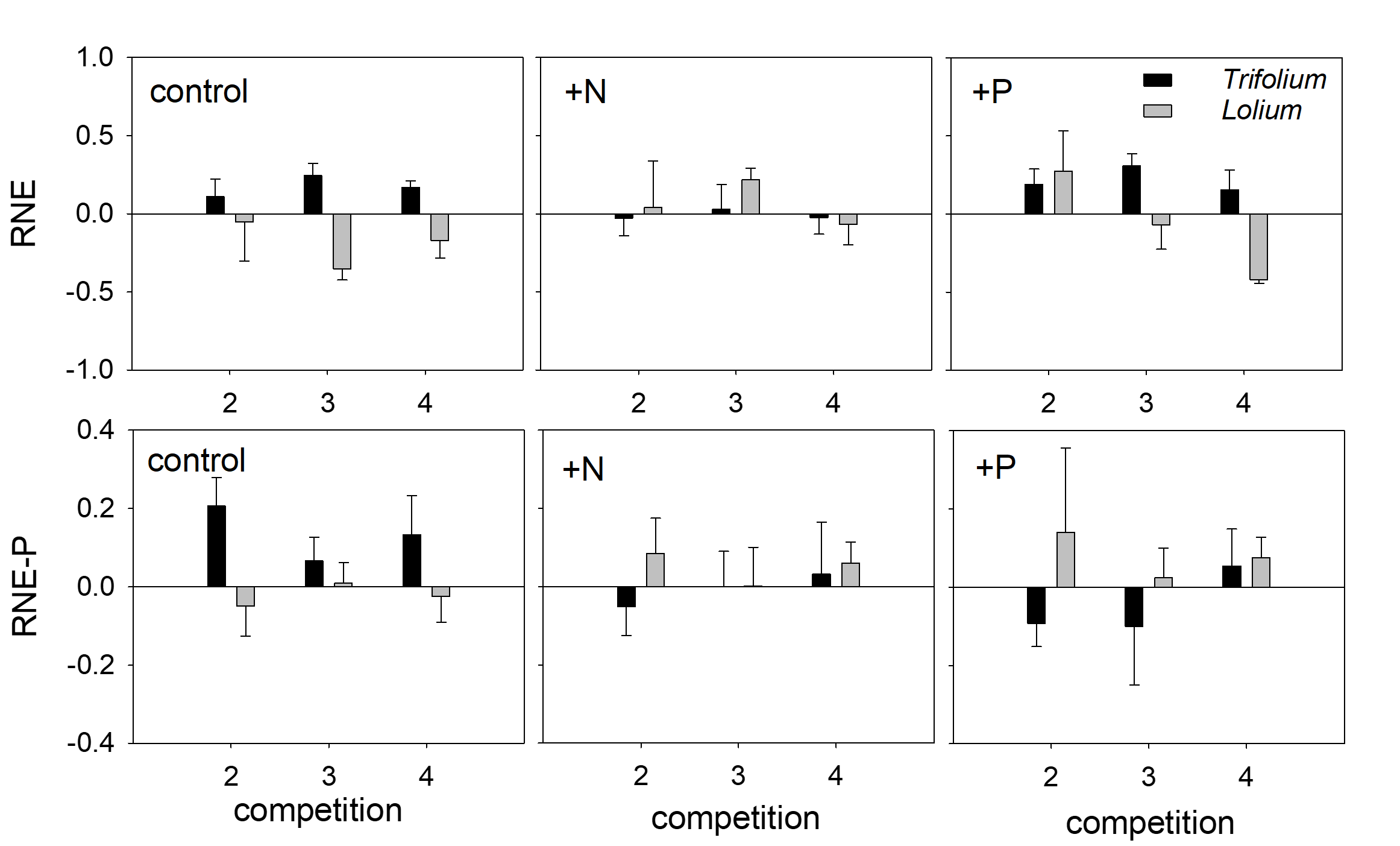

Supplement: Supplementary file 3 [file Image_2.TIF]
